# Spatiotemporal Analysis of Molecular Continuity in Pathogenesis of Squamous Cell Carcinoma: From Normal Epithelium to Precancerous Lesions and Carcinoma

**DOI:** 10.34133/cancomm.0051

**Published:** 2026-10-19

**Authors:** Lan Lan, Xinyu Wang, Xiaoqi Jiang, Xiangyi Lin, Yancheng Lai, Wen Tan, Shaosen Zhang, Dongxin Lin, Chen Wu

**Affiliations:** ^1^ Department of Etiology and Carcinogenesis, National Cancer Center/National Clinical Research Center/Cancer Hospital, Chinese Academy of Medical Sciences and Peking Union Medical College, Beijing, P. R. China.; ^2^ Key Laboratory of Cancer Genomic Biology, Chinese Academy of Medical Sciences and Peking Union Medical College, Beijing, P. R. China.; ^3^ Collaborative Innovation Center for Cancer Personalized Medicine, Nanjing Medical University, Nanjing, Jiangsu, P. R. China.; ^4^ Chinese Academy of Medical Sciences (CAMS) Oxford Institute, Chinese Academy of Medical Sciences, Beijing, P. R. China.

## Abstract

Squamous cell carcinomas (SCCs) develop from the stratified epithelia of the esophagus, head and neck, lung, and skin, providing a tractable model to investigate how altered epithelial fields progress toward invasive cancer. Across SCC subtypes, evolution often begins before overt malignancy, with age- and exposure-associated mutant clones expanding within histologically normal epithelia. As lesions advance, early driver selection becomes coupled to copy number alterations, chromosomal instability, and epigenetic remodeling, whereas later stages are shaped by branching diversification, cellular plasticity, and regionally constrained selection. Despite tissue-specific differences, several recurrent principles emerge, including clonal expansion within mutated normal fields, transition bottlenecks marked by genome-scale instability, and the spatial organization of subclonal architecture and invasive-front states. In this review, we synthesize recent progress in the temporal ordering and spatial patterning of SCC evolution and discuss how genetic alterations, epigenetic remodeling, and tissue ecology jointly drive disease progression. We further highlight the implications for early interception, biomarker development, immune evasion, and evolution-informed prevention and therapy.

## Background

Cancer initiation and progression are associated with the progressive accumulation of genetic and epigenetic alterations that disrupt tissue homeostasis and may confer selective advantages on altered clones. Accumulating evidence suggests that oncogene activation and tumor-suppressor gene inactivation, together with diverse epigenetic changes, can sustain proliferative signaling, weaken growth control and cell death programs, and remodel cellular metabolism, thereby contributing to malignant transformation and progression [1,2]. At the same time, tumorigenesis is not solely determined by cell-intrinsic alterations. Cancer cells evolve within a dynamic tissue environment composed of adjacent epithelial cells, infiltrating immune populations, stromal fibroblasts, vascular structures, and extracellular matrix, and reciprocal interactions with these components can substantially influence clonal behavior and disease development [3].

Recent advances in single-cell and spatial analyses have provided increasingly detailed views of intratumor heterogeneity and branched evolution, linking clonal diversification to progression, therapeutic resistance, and treatment failure [4]. These developments have made it increasingly feasible to examine how molecular alterations, cellular plasticity, and local selective pressures are organized across lesion stages and tissue regions. Squamous cell carcinomas (SCCs), arising in the epithelia of the esophagus, head and neck, lung, and skin, provide a particularly informative setting in which to examine these processes. Although these tumors differ in anatomical site and exposure history, they share several broad features, including relatively high mutational burden, widespread copy number alteration (CNA), and frequent epigenetic dysregulation, often in the context of environmental influences such as tobacco, alcohol, or ultraviolet (UV) radiation [5].

However, current discussions of SCC biology are still often organized either by organ site or by separate layers of molecular change, making it more difficult to appreciate how these events relate to one another across disease stages. In particular, the continuity linking altered epithelial fields, precancerous lesions, invasive transformation, and later diversification has not always been synthesized within a unified framework. Figure 1 provides an overview of this spatiotemporal framework.

**Fig. 1.**
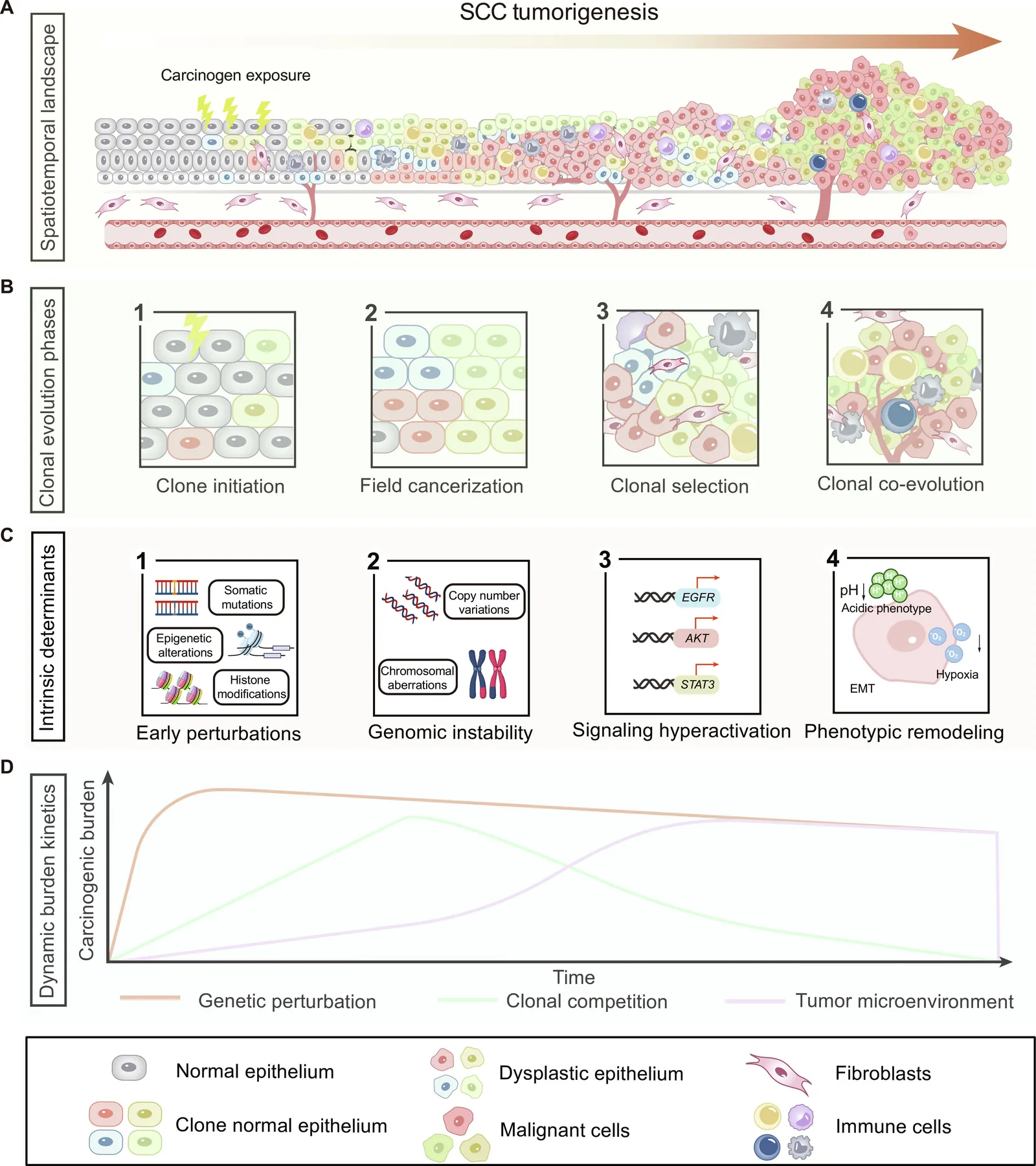
The spatiotemporal evolution and microenvironmental co-evolutionary process in SCC progression. (A) Spatiotemporal landscape of SCC tumorigenesis. The schematic illustrates the histological transition from normal epithelium to dysplastic epithelium and ultimately to malignant cells. This process is driven by continuous carcinogen exposure and is accompanied by progressive stromal remodeling, including the recruitment of fibroblasts and immune cells. (B) Sequential phases of clonal evolution. Clonal dynamics are categorized into 4 distinct stages: (1) clone initiation triggered by early mutational insults; (2) field cancerization characterized by the expansion of “mutated normal” clones; (3) clonal selection involving interclonal competition; and (4) clonal co-evolution between malignant populations and the surrounding niche. (C) Stage-specific intrinsic determinants. The molecular landscape evolves through (1) early perturbations, involving somatic mutations and epigenetic/histone modifications; (2) genomic instability, marked by CNVs and chromosomal aberrations; (3) signaling hyperactivation, particularly the up-regulation of the *EGFR, AKT,* and *STAT3* pathways; and (4) phenotypic remodeling, culminating in EMT, hypoxia, and an acidic metabolic phenotype. (D) Dynamic kinetics of carcinogenic burden. The line graph depicts the temporal fluctuations of key evolutionary drivers. Genetic perturbations (orange) peak early during initiation, followed by the rise of clonal competition (green) during the transition phase, and finally the maturation of the tumor microenvironment (pink) that sustains late-stage progression. Part of the schematic illustration is generated from BioRender (https://biorender.com/). SCC, squamous cell carcinoma; CNV, copy number variation; EGFR, epidermal growth factor receptor; AKT, protein kinase B; STAT3, signal transducer and activator of transcription 3; EMT, epithelial–mesenchymal transition.

In this review, we examine SCC progression from a spatiotemporal perspective, with emphasis on 4 interrelated themes: genetic and epigenetic alterations across precancerous and malignant stages, selective pressures imposed by the tumor microenvironment, advances in early detection and biomarker discovery, and emerging therapeutic strategies. By integrating these layers, we aim to provide a conceptual framework for understanding SCC development and progression, and to highlight opportunities for earlier interception and more precise prevention.

## An Integrated Conceptual Framework for SCC Evolution

Although SCCs arise in anatomically distinct stratified epithelia, including the esophagus, head and neck, lung, and skin, their development is not simply a juxtaposition of organ-specific events. Rather, it reflects a recurrent evolutionary logic that is reproduced across tissue contexts. At its core, cancer development is a Darwinian process of clonal evolution, in which heritable variation, differential fitness, and selection jointly shape disease trajectories [6–8]. A *“*clone” refers to a population of cells sharing a common ancestral alteration, whereas “clonal selection” describes the preferential expansion of those clones that gain a fitness advantage under local tissue constraints. In this setting, “fitness” is not a fixed property of a mutation itself, but the context-dependent capacity of a clone to survive, proliferate, compete, and persist within a given epithelial and microenvironmental niche [3,7,8]. These evolutionary relationships can be represented in a phylogenetic framework, in which “trunk” alterations denote early events shared by most or all tumor cells, whereas “branch” alterations arise later in restricted subclonal populations and underlie intratumor heterogeneity [9].

Current evidence indicates that SCCs do not emerge through a simple linear accumulation of scattered driver mutations, but through a continuous process shaped by early mutant clonal colonization, spatially constrained subclonal diversification, multilayer genomic disruption, convergence on shared pathway hubs, and persistent ecological selection. From this perspective, temporal progression, spatial diversification, and microenvironmental selection are best interpreted as coupled manifestations of a single multiscale evolutionary system rather than as separable phenomena [3,10,11].

### Early mutant clones colonize histologically normal epithelia

SCC evolution often begins in histologically normal epithelia, rather than being abruptly initiated only after overt premalignant lesions arise. In self-renewing epithelial tissues, somatic mutations accumulate with age, and a subset of mutant cells acquires a competitive advantage that enables clonal expansion while preserving overall tissue architecture [6,7,12]. Evidence from normal squamous epithelia, particularly the esophagus, supports this principle and indicates that mutant clonal expansion can precede overt dysplasia, thereby establishing an early evolutionary substrate for SCCs rather than a biologically neutral background [5,12–14].

From this perspective, “field cancerization” should not be understood merely as a pathological description of a broadly altered epithelial area, but as an evolutionary continuum linking normal epithelium, precancerous lesions, and invasive carcinoma. Originally introduced as a morphological concept and later reformulated in molecular and evolutionary terms, field cancerization describes geographically extended epithelial areas that harbor widespread molecular alterations and competing clones before overt malignant transformation [10,11,15,16]. In SCCs, it is therefore best regarded as the tissue-level manifestation of early clonal evolution and the substrate from which later diversification and progression emerge [12,13].

### Spatial constraint governs branch diversification

Once clonal expansion has established an abnormal epithelial substrate, subsequent evolution does not proceed freely within a homogeneous background but instead unfolds under strong spatial constraint [7,9,17,18]. In phylogenetic terms, trunk events denote early alterations shared across sampled tumor regions, whereas branch events are confined to subsets of regions or subclones and reflect subsequent diversification during tumor evolution [9,19–21]. In SCCs, however, the trunk-branch framework is not purely temporal. Branch diversification is intrinsically spatial, because subclonal expansion is constrained by local tissue structure, anatomical compartmentalization, and microregional differences in selective pressure [20,22]. The persistence, expansion, or extinction of subclones depends on the ecological niches they occupy and on their ability to adapt to local selective regimes [17,23]. Accordingly, spatial heterogeneity in SCCs is not merely a projection of temporal heterogeneity; rather, it reflects the regional organization of clonal architecture, copy number states, and local selective pressures [9,20]. Thus, branch diversification in SCCs should be understood as a regionally structured process in which local tissue architecture and ecological context continuously shape phylogenetic trajectories [3,24–27].

### Genome-wide disruption accelerates SCC evolution

Large-scale cancer genome studies have established a multilayered framework for interpreting tumor evolution. Across human cancers, evolutionarily relevant genomic alterations extend far beyond coding single-nucleotide variants (SNVs) and small insertions/deletions (indels), and include noncoding driver events, somatic CNAs (SCNAs), structural variants (SVs), chromosomal instability (CIN), localized hypermutation phenomena such as kataegis, and whole-genome doubling (WGD) [28–31]. This broader view is particularly important in SCCs, in which the tempo of progression is often shaped not only by the number of mutated genes but also by the emergence of genome-scale plasticity.

SCNAs comprise focal or broad gains and losses of genomic material that alter oncogene and tumor-suppressor dosage, thereby generating immediate pathway-level consequences [32,33]. CIN refers to the persistent mis-segregation and structural reorganization of chromosomes, creating ongoing karyotypic diversity on which selection can act, whereas WGD is increasingly recognized as a macro-evolutionary event that buffers deleterious alterations, increases tolerance to aneuploidy, and permits subsequent rounds of chromosomal diversification [34–37]. More broadly, complex SV processes including chromothripsis-like events, chromoplexy, and breakage–fusion–bridge (BFB) cycles can restructure the genome through catastrophic, punctuated, or iterative episodes rather than solely through gradual accumulation [36,38–40]. Collectively, these observations indicate that malignant progression is driven not by point mutations alone but by interacting lesions operating across gene-scale and chromosome-scale levels [29,41–44].

A second major advance from cancer genome studies is the definition of “mutational signatures” as reproducible patterns of base substitutions, doublet substitutions, and small indels that function as molecular readouts of DNA damage and DNA repair history [45–47]. These signatures include exposure-associated processes, such as tobacco-related single-base substitution signature 4 (SBS4) and UV-related SBS7, as well as endogenous processes linked to defective DNA repair or dysregulated enzymatic activity, including homologous recombination deficiency-associated SBS3, mismatch repair-related signatures, polymerase-associated signatures, and SBS2 and SBS13 associated with the apolipoprotein B mRNA-editing enzyme, catalytic polypeptide-like (APOBEC) family [47–51]. In this framework, genomic instability and mutational signatures are complementary rather than separate concepts: The former describes the scale and structure of genomic disruption, whereas the latter helps infer the processes that generated it. This distinction is especially relevant to SCCs, because different anatomical SCCs combine relatively high mutational burden with substantial copy number complexity, yet remain shaped by distinct dominant mutagenic exposures and repair states [52,53].

This principle is particularly evident in esophageal squamous cell carcinoma (ESCC). Integrated genomic studies of ESCC have shown that recurrent driver events coexist with frequent CNAs and structural complexity, suggesting that ESCC evolution is shaped not simply by the accumulation of additional mutations but by layered genomic alterations that may enlarge the pool of selectable variants and adaptive trajectories [54–57]. Crucially, however, this expanding genomic diversity does not translate into unlimited functional diversity. Instead, distinct mutational processes and genome-scale alterations repeatedly converge on a restricted set of biologically consequential pathway hubs.

### Distinct mutational routes converge on shared pathway hubs

Although SCCs arising at different anatomical sites are shaped by distinct mutagenic environments, their molecular evolution does not resolve into completely separate, organ-specific patterns. Rather, a recurring principle emerges from large-scale cancer genome studies: Genetically diverse lesions repeatedly converge on a limited set of core functional pathways. Thus, the importance of genome-wide disruption lies not only in the diversity it generates but also in the recurrent tendency of selection to channel that diversity toward a relatively restricted set of biologically consequential outputs. This is consistent with pan-cancer observations showing that, despite diverse gene-level alterations, tumor evolution repeatedly converges on a restricted set of modules, including cell cycle control, squamous differentiation programs, phosphoinositide 3-kinase/receptor tyrosine kinase (PI3K/RTK) signaling, oxidative-stress responses, and chromatin-regulatory networks [5,29,30,58]. ESCC provides direct evidence for this model of pathway convergence with mutational heterogeneity. In ESCC, recurrent genetic lesions arising through distinct mutational processes repeatedly converge on cell cycle, differentiation, chromatin-regulatory, and immune-relevant programs [21,56,59]. Taken together, these findings suggest that the functional output of ESCC evolution is not random genomic complexity per se, but repeated perturbation of a bounded set of lineage- and fitness-relevant pathways.

Head and neck squamous cell carcinoma (HNSCC) and cutaneous squamous cell carcinoma (cSCC) reinforce this principle from distinct mutagenic backgrounds. The Cancer Genome Atlas (TCGA) and related studies show that HNSCC remains strongly stratified by human papillomavirus (HPV) status, whereas cSCC carries the heavy mutational imprint of UV exposure; yet, in both settings, selection still converges on a limited set of lineage-relevant and fitness-relevant pathways [52,60,61]. In cSCC, UV-associated SBS7a, SBS7b, SBS7c, and SBS7d—signatures linked to UV-induced pyrimidine-dimer damage—provide a direct mechanistic link between exposure history and the mutational landscape, while comparative analyses further indicate that different exposure contexts are associated with distinct mutational processes [47,60]. Accordingly, comparisons across ESCC, HNSCC, and cSCC should not be read merely as parallel displays of site-specific molecular atlases. Rather, they represent alternative realizations of the same evolutionary principle in different tissue contexts: UV radiation, tobacco, alcohol, viral infection, and endogenous APOBEC activity can each perturb similar pathway hubs through distinct molecular routes, thereby producing SCC evolutionary trajectories that share a common biological core while retaining exposure-specific molecular imprints [47,52,60,61].

### Clonal fitness emerges from genetics, plasticity, and ecology

Clonal success is not determined by genomic alteration alone. The phenotypic consequence of the same lesion depends strongly on lineage context, epigenetic state, and environmental constraint such that identical or closely related genomic alterations may generate divergent evolutionary outcomes across lesions, anatomical sites, and disease stages [17,23,62,63]. In this sense, clonal fitness is not a single-gene property, but an emergent systems-level attribute arising from the interaction among genetic lesions, cell-state plasticity, and tissue ecology [7,8,64,65]. Contemporary evolutionary models further emphasize that fitness is continuously shaped across time and space by reciprocal coupling between genotype and cellular and ecological contexts, rather than by mutation status alone [66,67].

Accordingly, the microenvironment should therefore not be regarded as a passive backdrop to SCC evolution, but as an active contributor to tumor survival and progression [68–71]. Under chronic injury, inflammation, and regenerative pressure, ecological conditions can shift the selective value of variants that would otherwise remain neutral or constrained, while immune, stromal, vascular, and metabolic factors further reshape local fitness landscapes [72–75]. Thus, the selective value of a lesion is neither fixed nor monotonic but depends on how effectively a given clone is matched to the ecological conditions in which it evolves.

A further implication is that genotype-to-phenotype translation itself is plastic. Whether a clone persists and ultimately progresses depends on the interaction between genetic alterations, cellular-state programs, and supportive stromal and immune niches [76,77]. This process may be broadened by reversible plasticity, stress-adaptation programs, and nongenetic memory; in some settings, these nongenetic states may precede or coexist with more stable genetic alterations [65,78–80]. In SCCs, this issue is especially relevant because chronic damage and fluctuating inflammatory conditions can alter the selective interpretation of the same genomic lesion.

## Spatial Organization of Tumor-Intrinsic Heterogeneity in SCCs

Spatial heterogeneity in SCCs should not be regarded merely as a passive projection of preexisting molecular differences onto histological sections. Rather, it represents a fundamental organizational principle of tumor-intrinsic evolution itself. Whereas the temporal dimension primarily addresses the order in which evolutionary events arise, the spatial dimension concerns how distinct clones, copy number states, and tumor cell states become regionally organized within SCC lesions, with implications for local invasion, metastatic dissemination, and therapeutic stratification [19,21,81,82]. Broadly, the spatial architecture of SCCs can be understood at 3 interrelated levels: region-specific clonal and copy number architecture, field geometry shaped by directional expansion, and local reprogramming of tumor cell states at the invasive front.

### Region-specific clonal and copy number architecture

A recurrent feature across SCCs is the presence of multiple spatially segregated, phylogenetically related, yet clearly diverged subclonal populations within the same lesion. In other words, SCC progression can involve the parallel evolution of multiple locally related branches rather than the homogeneous expansion of a single genome [9,19–21].

ESCC provides some of the clearest evidence for this principle. Multi-region sampling and phylogenetic reconstruction have shown that ESCC is dominated by branched evolution, whereas linear evolution is comparatively uncommon [83]. Distinct compartments of the primary tumor—particularly superficial versus deeper subregions—as well as matched lymph node metastases can display region-specific mutation profiles and copy number architectures, indicating that even tumor cells derived from a common ancestral clone may continue to diverge along local branches within distinct spatial compartments [84]. Such regional specificity is not confined to point mutations. High-resolution analyses have further demonstrated near-universal cell cycle dysregulation in ESCC, with inactivation of *CDKN2A* (cyclin-dependent kinase inhibitor 2A) and amplification of cyclin D1 (*CCND1*) observed in 76% and 57% of tumors, respectively; at the same time, novel focal deletions involving vestigial like family member 4 (*VGLL4*) and autophagy related 7 (*ATG7*) at 3p25.2 suggest that Hippo- and autophagy-related alterations may also contribute to the genomic architecture of ESCC [85]. Although gain of 11q13 represents a relatively common canonical genomic hallmark in ESCC, copy number states themselves remain regionally heterogeneous, indicating that shared trunk features and local branch diversification can coexist within the same tumor [20]. The resulting highly branched clonal organization has direct clinical implications: Single-site biopsies may fail to capture the full extent of subclonal complexity, including alterations restricted to individual tumor regions [83].

Comparable regionally branched architectures are also observed in HNSCC, particularly oral squamous cell carcinoma (OSCC), and in lung squamous cell carcinoma (LUSC). Evolutionary tree analyses have shown that subclones within the same murine lymph node, although independently seeded from the primary tumor, can continue to diverge into distinct branches after dissemination, with oncogenic mutations in *SWI*/*SNF* (switch/sucrose nonfermentable)-related, *Smarca4* (switch/sucrose nonfermentable-related, matrix-associated, actin-dependent regulator of chromatin, subfamily A, member 4), *Cxcr4* (C–X–C motif chemokine receptor 4), *Ctnnd2* (catenin delta 2), and *Ank1* (ankyrin 1) implicated as drivers of this process [86]. This finding indicates that metastatic lesions are not static replicas of the primary tumor, but instead dynamic clonal populations that continue to evolve within newly occupied spatial compartments. In LUSC, this pattern is further reflected in regional divergence driven by persistent CIN. A subset of LUSCs exhibits arm-level parallel evolution, as evidenced by mirrored subclonal allelic imbalance; moreover, key oncogenes such as *CDK4* (cyclin-dependent kinase 4) and *BCL11A* (BCL11 transcription factor A) may undergo independent focal amplification in distinct subclones, reflecting convergent responses to similar adaptive demands in different local regions [19]. Spatial heterogeneity in SCCs, therefore, should first be understood as the organized regional distribution of distinct clonal and copy number architectures.

### Field geometry and directional clonal expansion

Beyond local subclonal branching, the spatial organization of SCCs is also shaped by larger-scale field effects and directional clonal expansion. In other words, spatial heterogeneity does not arise only after tumor formation, but is prefigured much earlier by tissue architecture and the geometry of clonal spread [10]. This is especially evident in the esophagus. Clonal populations in the normal esophageal epithelium exhibit striking macroscopic expansion, extending across hundreds of micrometers—far beyond the crypt-restricted clonal units characteristic of the colon—and thereby generating a broader, potentially pro-tumorigenic field [87]. In ESCC, a “spatially directed evolution” model derived from multi-region analysis further suggests that subclones may display biased expansion toward the upper esophagus [88]. Thus, the spatial pattern of ESCC is not merely the result of stochastic regional variation but instead reflects a process of clonal reorganization governed by directionality and geometric constraint.

A similar phenomenon, in which spatial geometry precedes overt tumor formation, is also observed in LUSC. Experimental lineage-tracing studies have shown that LUSC may originate from airway basal cells; following non-neutral clonal competition, these cells actively migrate along the bronchial tree from proximal to distal airways and give rise to clonally related premalignant lesions across distinct anatomical lobes [89]. This finding indicates that the multifocality and regional relatedness of lung SCCs are not simply consequences of late dissemination, but may instead be rooted in the early spatial expansion of abnormal clones along anatomical routes. More generally, evolutionary modeling and spatial growth analyses have also emphasized that clonal spread is constrained by tissue topology and region-specific ecological opportunity rather than by purely stochastic drift [27,90]. Transgenic lineage tracing further demonstrates that the dynamics of clonal competition are strongly influenced by the surrounding cellular environment [91]. Thus, whether in the form of large-scale clonal spread in the esophagus or abnormal migration along the bronchial tree in the lung, the spatial architecture of SCCs reflects clear field geometries and non-neutral expansion trajectories.

### Spatial organization of tumor cell states at the invasive front

In addition to clonal and copy number architecture, the spatial heterogeneity of SCCs is also manifested in the localized organization of tumor cell states, particularly at the invasive front. The invasive front is not merely a pathological boundary; rather, it represents a spatial site at which tumor cell plasticity, local adaptation, and functional output are most intensely concentrated. More broadly, single-cell studies of HNSCC have shown that a partial epithelial-to-mesenchymal transition (p-EMT) program enriched for extracellular matrix-related genes localizes to the leading edge of primary tumors and is associated with invasion and metastasis [81].

cSCC provides especially clear evidence for this level of organization. Multimodal profiling of human cSCC has identified tumor-specific keratinocytes (TSKs), a cancer-specific keratinocyte population localized within fibrovascular niches at tumor leading edges, where they exhibit regulatory programs associated with activator protein 1 (AP-1) and E26 transformation-specific (ETS) factors, EMT-like features, extracellular matrix remodeling activity, and extensive ligand–receptor communication with stromal and immune cells [82]. During the transition from normal skin to premalignant lesions, markers such as matrix metallopeptidase 10 (*MMP10*) already display striking spatial specificity, while fatty acid metabolism-related genes are progressively down-regulated, indicating that state reprogramming at the invasive front is not a late incidental phenomenon but a spatial program progressively established during early malignant transformation [82,92,93]. In spatial analyses of SCCs, the invasive front should therefore be viewed as a key functional site of tumor cell-state heterogeneity rather than as a mere histological margin.

Beyond transcriptional cell-state organization, spatial heterogeneity at the invasive front may also reflect regional variation in the mutational processes shaping tumor evolution. From the perspective of mutational processes, cSCC is strongly shaped by UV-associated DNA damage, as reflected by single-base substitution signatures SBS7a, SBS7b, SBS7c, and SBS7d, as well as by doublet-base substitution signature 1 (DBS1), which is typified by CC>TT transitions [47]. In addition, APOBEC-associated mutagenesis, with enrichment of SBS2/SBS13, may further contribute to mutational diversification in selected tumors [47,60]. Here, APOBEC refers to the endogenous family of cytidine deaminases whose dysregulated activity can generate characteristic cytosine mutations in single-strand DNA. This mutational process perspective indicates that heterogeneity in SCC not only is a matter of different clones occupying different regions but also reflects the coexistence of distinct mutagenic processes and cell-state programs.

### Microenvironmental forces that shape spatial evolution

Although tumor-intrinsic clones, copy number architectures, and cell states represent the direct manifestations of spatial heterogeneity, these patterns do not arise in a vacuum. Local microenvironmental factors—including physical geography, exposure gradients, hypoxia, immune editing, and stromal cellular components—impose persistent selective pressures upon preexisting spatial architectures and thereby shape regional clonal fate and functional output [68–70,94]. In ESCC, spatial selection is first manifested through physical and anatomical constraints. The biased expansion of subclones toward the upper esophagus has been proposed to reflect local physical geography and differential exposure to food-related mechanical stress [88]. In addition, lineage-tracing studies have shown that clonal expansion and competition are strongly influenced by the surrounding cellular environment [91], suggesting that even after tumor-intrinsic branches have formed, local context continues to participate in clonal selection. Spatial transcriptomic analyses have further revealed aberrant epithelial–macrophage communication in ESCC, with the macrophage migration inhibitory factor (MIF)–CD74 receptor axis showing potential pro-tumor interactions between macrophages and epithelial cells, suggesting that macrophage-associated ecological niches may contribute to local tumor-state diversification [95]. Thus, the spatial diversification of ESCC may reflect not only tumor-intrinsic differences but also the contribution of macrophage-associated ecological niches.

In HNSCC, particularly OSCC, local hypoxia and immune pressure likewise reshape spatial evolution. Phylogenetic reconstruction in an OSCC mouse model suggests that disseminated tumor cells and primary lesions can follow distinct evolutionary trajectories, implying that node-specific microenvironmental selective pressures may shape branch diversification [86]. In parallel, invasive progression can be facilitated by the recruitment of bone marrow-derived cells that provide extracellular matrix remodeling proteases and thereby contribute to basement membrane remodeling, angiogenesis, and local niche reorganization. Consistent with this view, in a multistage mouse model of cutaneous SCC, bone marrow-derived inflammatory cells were identified as important sources of matrix metalloproteinase 9 (MMP-9), which contributed to keratinocyte hyperproliferation, angiogenesis, and malignant progression [96]. In addition, tumor necrosis factor (TNF) appears to play temporally distinct roles during tumor evolution: the *Nature* 2024 study by Renz et al. [77] showed that TNF signaling supported early clonal expansion, whereas invasive cancer cells later switched to an autocrine TNF program associated with epithelial–mesenchymal transition, a distinct tumor state, and adverse clinical outcome. In LUSC, microenvironmental shaping is reflected in the coupling between local clonal architecture, neoantigen landscapes, antigen presentation status, and immune selection. Local mutational diversity has been linked to intratumoral immune heterogeneity, while neoantigen loss, human leukocyte antigen (HLA) alterations, and antigen presentation defects accompany immune escape during lung cancer evolution [97,98]. Together, these findings suggest that clonal architecture and immune selection are coupled during lung cancer evolution, and that similar immune–genomic coupling may contribute to microenvironmental heterogeneity in LUSC.

In cSCC, microenvironmental influence is likewise manifested through the superimposition of local exposure and local ecological niches. UV-exposed skin can accumulate and select for mutant keratinocyte clones in a body site-dependent manner, indicating that local exposure history and tissue geography help shape the preexisting mutant epithelial field from which cSCC may arise [99]. In addition, TSKs are primarily localized within fibrovascular microenvironments [82], indicating that spatial reprogramming at the invasive front is not simply a tumor-intrinsic program but is tightly coupled to local stromal ecological support. In other words, the distinction among spatial regions in cSCC records not only mutational history but also the combined influence of local exposure and stromal support.

Taken together, spatial evolution in SCCs can be resolved into 2 interconnected levels. First, tumor-intrinsic spatial heterogeneity is manifested as region-specific clonal branching, copy number architectures, field geometric expansion, and invasive-front cell-state reprogramming; these features determine where particular clones reside within lesions and where specific functional programs are deployed. Second, local microenvironmental factors—including physical geographic constraints, exposure gradients such as UV radiation or food contact, hypoxia, immune editing, and stromal cellular components—are not secondary additions to these patterns, but act upon preexisting spatial differences to determine which branches are amplified, which states are maintained, and which regions are more likely to become functional niches for invasion and therapeutic resistance. Spatial heterogeneity in SCCs should therefore not be understood as a static difference across tissue sections, but as the spatialized outcome of sustained interactions between tumor-intrinsic evolution and local microenvironmental selection.

## Temporal Dynamics of Tumor Evolution in SCCs

The temporal evolution of SCCs follows a hierarchical and continuous trajectory. It begins with the long-term accumulation and competitive expansion of mutant clones in histologically normal epithelia; trunk drivers are then progressively fixed during premalignant progression and become coupled to CNA and CIN, enabling lesions to cross the threshold into malignancy; once invasion is established, subclonal branches acquire additional adaptive advantages and may undergo punctuated acceleration through mechanisms such as extrachromosomal DNA (ecDNA), BFB cycles, or WGD. SCC evolution should therefore not be viewed as a simple projection of pathological stage, but rather as a dynamic sequence encompassing competition in normal epithelium, trunk establishment, genome-scale acceleration, and late branch adaptation [6,7,100]. Here, the emphasis is placed on the order in which altered clones arise, are selected and fixed, and subsequently diversify, rather than on their regional distribution within lesions. This temporal framing is especially useful in SCCs because many canonical drivers can already be detected in histologically normal or premalignant epithelia, whereas the transition to invasion is often marked by a later surge in genome instability, structural complexity, and branch-specific adaptation [12,100].

### Early mutant clones accumulate and compete before overt malignancy

A central principle of SCC evolution is that key evolutionary processes begin in histologically normal epithelium, rather than being initiated after the appearance of overt premalignant or invasive lesions. The esophagus provides some of the clearest evidence for this principle. In normal esophageal epithelium, somatic mutations accumulate continuously with age; quantitative sequencing studies have shown that normal esophageal cells acquire on average approximately 40 mutations per cell per year, with notch receptor 1 (*NOTCH1*)*,* notch receptor 2 (*NOTCH2*)*,* and tumor protein p53 (*TP53*) among the most frequently mutated genes; by midlife, this gradual accrual gives rise to a patchwork of mutant clones covering much of the esophageal epithelium, thereby creating a fertile substrate for subsequent malignant selection [13]. Subsequent studies further showed that positively selected clones occupy less than 10% of the epithelium in individuals in their 20s, but may remodel as much as 80% of the epithelial compartment in those older than 60 years [14]. Together, these observations indicate that normal epithelium is not a neutral background for later tumorigenesis, but rather a competitive mutant clonal ecosystem maintained through selection and replacement [12].

Functional studies further suggest that early clonal competition is governed not by a single driver, but by a set of key regulators that jointly shape cell fate, tissue fitness, and growth advantage, among which *TP53*, phosphatidylinositol-4,5-bisphosphate 3-kinase catalytic subunit α (*PIK3CA*), and *NOTCH1* are particularly prominent [101–103]. Sequencing of mutagen-exposed mouse esophageal epithelium revealed strong positive selection for mutations in *Notch1*, *Notch2*, and *Trp53*, indicating that these alterations can reshape clonal competition before overt tumor formation [91]. Mechanistically, loss of Notch signaling alters the balance of cell fate by reducing symmetric differentiation divisions, thereby conferring sustained expansion capacity on mutant clones; these clones not only expand through intrinsic fitness advantages but also promote the differentiation and loss of neighboring wild-type cells from the tissue, thereby facilitating colonization of the local epithelium [104]. Clustered regularly interspaced short palindromic repeats (CRISPR)-based functional screening and pharmacologic perturbation further showed that *PIK3CA* mutations enhance the competitive fitness of mutant cells in normal esophageal epithelium through activation of the PI3K–mTOR (mechanistic target of rapamycin) pathway, underscoring that metabolic and growth signaling can shape clonal fate already at the premalignant stage [102]. Conceptually, these observations align with broader evolutionary models in which early tumorigenesis reflects non-neutral competition among clones that differ in fitness, differentiation bias, and tissue adaptability [17].

Importantly, early clonal competition is not necessarily tumor-promoting. Multiple studies have shown that highly fit “mutant-normal” clones can actively eliminate incipient microscopic tumor foci through endogenous cell competition; this process is notably independent of immune surveillance and is proposed to occur through displacement of the lesion from the basal layer, followed by extrusion and shedding [73,91]. Thus, the mutant clonal landscape of normal epithelium can serve both as the evolutionary substrate for later carcinogenesis and, at specific stages, as an intrinsic barrier to malignant progression [12,14,73]. In parallel, the development of a 3-dimensional esophageal epithelioid system has provided a powerful platform for long-term investigation of clonal competition, selection, and cell dynamics [105]. Similar logic has also been proposed in broader epithelial evolution frameworks, in which mutant clone expansion, competition, and replacement are understood as dynamic and context-dependent rather than linearly tumor-promoting [10].

Comparable early temporal architectures are also observed in other SCCs. In a murine model of oral carcinogenesis, lineage tracing showed that normal oral epithelial progenitors can be reprogrammed into tumor-initiating stem-like cells under spatiotemporally controlled activation of oncogenes and inhibition of tumor suppressors, demonstrating that premalignant fate conversion can be resolved at single-cell resolution [106]. In the skin, chronic UV exposure similarly promotes mutant keratinocyte fields enriched for cSCC-associated driver alterations, including *TP53*, *NOTCH1*, and FAT atypical cadherin 1 (*FAT1*) [60,99]. As in the esophagus, however, these early clones do not expand without limit. The epidermis imposes homeostatic constraints that partially restrain their growth. More specifically, expansion of *TP53*-mutant clones is not driven solely by proliferative advantage, but also by altered tissue mechanics and cell adhesion; the dynamics of *TP53*-mutant clones are shaped not only by proliferative advantage but also by tissue adaptation and cell competition [107]. Thus, the early phase of cSCC evolution is not a linear expansion of a single dominant clone but a dynamic process of replacement and reselection. By contrast, clonal selection in normal human skin varies markedly with body site and exposure context, highlighting the key role of environmental stress—particularly UV irradiation—in accelerating this early temporal process [99]. In oral premalignant lesions, analogous evidence is emerging that genetically altered precursor fields preceded overt carcinoma, further supporting the idea that SCCs arise from already selected epithelial territories rather than genetically naive mucosa [108].

### Premalignant fixation of trunk drivers and progressive genomic instability

Following the establishment of early mutant clonal fields, SCC evolution enters a second temporal layer in which trunk drivers become progressively fixed and are coupled to genome instability during premalignant progression, thereby building the genetic framework for malignant transformation. ESCC provides the most complete illustration of this stage. Large-scale whole-genome sequencing of 508 ESCC cases expanded the genomic landscape of this disease and identified *TP53* (74.8%) as the most frequently mutated candidate driver, followed in frequency by *FAT1* (15.9%), *NOTCH1* (15.4%), and lysine methyltransferase 2D (*KMT2D*) (14.8%); beyond these recurrent drivers, high-resolution genomic analysis also highlighted a specific role for the NFE2-like bZIP transcription factor 2 gene (*NFE2L2*) and its encoded nuclear factor erythroid 2-related factor 2 protein (NRF2) in ESCC evolution [109]. In ESCC, *NFE2L2* alterations, including mutations affecting the DLG/ETGE motifs that disrupt the degradation of NRF2 mediated by Kelch-like ECH-associated protein 1 (KEAP1), are associated with increased nuclear NRF2 activity; together, high nuclear NRF2 expression and *NFE2L2* alterations predict poor prognosis [110]. This suggests that some drivers do not fit neatly into simple tumor-suppressor loss or oncogene activation categories, but instead alter stress-response thresholds and survival programs to provide additional adaptive capacity during disease progression.

Phylogenetic reconstruction indicates that driver mutations are preferentially truncal, although approximately 40% are branched or subclonal. Half of the branched driver mutations involve oncogenic alterations such as *PIK3CA*, *NFE2L2*, and mechanistic target of rapamycin kinase (*MTOR*), whereas most truncal driver mutations affect tumor-suppressor genes such as *TP53*, *KMT2D*, and zinc finger protein 750 (*ZNF750*) [20]. Consistently, PhylogicNDT-based timing analyses showed that *TP53* and *CDKN2A* are typically early drivers, whereas *NFE2L2*, *SMARCA4*, *PIK3CA*, and *CUX1* tend to arise later [88]. This ordering is particularly important during the transition from normal epithelium to premalignant lesions. *TP53* mutations can be fixed by clonal selection in the premalignant background. In transgenic mouse models, induction of *Trp53* mutation in normal esophageal progenitors confers a proliferative advantage and clonal expansion while preserving overall epithelial integrity; by contrast, complete loss of functional *Trp53* alleles triggers aneuploidy and copy number aberrations, marking a transition from relative genomic stability to instability [101]. More broadly, this pattern is consistent with the concept that trunk establishment often precedes the major surge in chromosome-scale disruption so that the key bottleneck from precursor to cancer lies less in the first appearance of drivers than in the subsequent acquisition of sufficient genomic plasticity to sustain malignant expansion [34,111].

Notably, early driver events do not necessarily exert uniform effects across all stages of disease progression. Systematic analyses of ESCC-associated drivers indicate that although *NOTCH1* and *TP53* mutations confer strong selective advantages in normal epithelium, their subsequent roles are highly context-dependent [101,103]. In particular, despite the high frequency of *NOTCH1* alterations in histologically normal epithelium, emerging evidence suggests that under specific conditions *NOTCH1* alterations may suppress tumor growth, helping explain why certain *NOTCH1* mutations are more frequent in normal tissue than in invasive ESCC [103]. To resolve this apparent paradox, recent work has proposed that aberrant Notch signaling becomes critical for tumor angiogenesis during invasive transition; unlike competitive clones in normal epithelium, malignant cells may retain normal NOTCH1 signaling through the NOTCH1–USP5–STAT3 axis to support vascularization and sustained growth, which may help explain the lower frequency of *NOTCH1* mutations in invasive ESCC [112]. Accordingly, the role of *NOTCH1* in SCC evolution cannot be reduced to a simple early driver or late suppressor, but is better understood as a context-dependent regulatory node whose function shifts with tissue setting and disease stage.

Consistent with this model, the average number of arm-level CNAs rises progressively from 0.2 per clone in normal epithelium to 1.4 in low-grade intraepithelial neoplasia (LGIN), 6.6 in high-grade intraepithelial neoplasia (HGIN), and 13.8 in ESCC [111]. Gain of chromosome 3q, particularly involving tumor protein p63 (*TP63*) and SRY-box transcription factor 2 (*SOX2*), has been proposed as a key switch that drives progression toward intraepithelial neoplasia [113]. Thus, the central process at the premalignant stage is not simply the appearance of more mutations, but the progressive coupling of trunk driver fixation to chromosome-level instability. This interpretation is consistent with broader cancer-genome studies showing that SCNAs, CIN, and, in some tumors, later WGD provide major substrates for selection by increasing dosage imbalance, karyotypic diversity, and adaptive plasticity [32,33,35,36].

A similar temporal structure is seen in HNSCC. HPV-negative tumors are largely driven by lifestyle-associated exposures and are characterized by early, frequent *TP53* mutation and *CDKN2A* loss; by contrast, HPV-positive tumors bypass this route through the viral oncoproteins E6 and E7, and instead display enrichment of *PIK3CA* mutations and loss of TNF receptor-associated factor 3 (*TRAF3*) [61,114]. Analyses of potentially premalignant oral lesions (PPOLs) further indicate that core driver alterations—including *TP53*, *CDKN2A*, *KMT2D*, *PIK3CA*, and *NOTCH1*—are already established at the premalignant stage [108,115–117]. In an OSCC mouse model, phylogenetic reconstruction further resolved ancestor-descendant relationships among tumor subclones [86], supporting a traceable branching process in which primary lesion-derived clones disseminate early and continue to diverge across tissue compartments. Broad SCNAs, however, do not necessarily arise synchronously with these early point mutations; instead, they tend to emerge once cells acquire immortality and escape senescence, marking a decisive evolutionary leap from relative genomic stability to highly unstable malignancy [117]. In this sense, HNSCC exhibits a marked temporal decoupling between point mutations and structural abnormalities [61,117–121]. This “transition bottleneck” model is also supported by recent syntheses arguing that the crucial step from premalignant lesion to invasive carcinoma often coincides with a sharp escalation in genome instability rather than with the simple additive accumulation of low-level mutation burden [122].

LUSC likewise displays a strongly hierarchical temporal architecture. Early foundational events include *NOTCH1* mutations, 3q amplification encompassing *SOX2* and *PIK3CA*, and losses of 3p, 4p, and 5q, and 17p; their predominantly clonal and pre-genome-doubling timing supports their involvement in tumor initiation [19]. Although *TP53* loss typically also occurs early, its functional disruption cooperates with CIN to accelerate tumor evolution and increase genomic complexity [19]. In cSCC, the temporal principle is expressed as early UV-induced tumor-suppressor disruption coupled to progressive dedifferentiation. Single-cell analyses indicate that *TP53* and *NOTCH1* alterations arise early during keratinocyte evolution and may prime cells for malignant transformation by increasing mutational burden and perturbing DNA damage control, apoptosis, and differentiation programs [123]. Within this continuum, promoter mutations in telomerase reverse transcriptase (*TERT*) emerge as early events at the actinic keratosis (AK) stage, whereas late inactivation of AT-rich interaction domain 2 (*ARID2*) followed by activation of the mitogen-activated protein kinase (*MAPK*) pathway defines the transition toward invasive behavior [123]. In addition, the gradual up-regulation of progenitor-like programs and down-regulation of late epidermal differentiation programs further indicate that temporal progression in cSCC is accompanied by sustained cell-state dedifferentiation [92].

### Late subclonal adaptation and catastrophic progression

Once the trunk architecture has been established, SCC evolution enters a third temporal layer characterized by late subclonal adaptation, therapy-relevant selection, and the accumulation of catastrophic genomic rearrangements. At this stage, progression cannot be explained solely by the acquisition of additional driver alterations. Rather, ongoing genomic diversification generates competing subclones, and microenvironmental or therapeutic pressures favor the expansion of lineages with fitness-enhancing combinations of genomic alterations and phenotypic traits. Conceptually, this phase represents selection-driven remodeling of the subclonal composition upon an established genetic scaffold and is often associated with treatment resistance and metastatic potential [18].

In ESCC, this is particularly evident. Multi-region sequencing analyses suggest that a substantial proportion of additional somatic mutations accumulate progressively after malignant transformation [21]. Recurrent mutations in erb-b2 receptor tyrosine kinase 4 (*ERBB4*) have been identified and functionally validated as oncogenic [21]. Early mutations in *BRCA1* and *BRCA2* are significantly associated with SBS3, a signature linked to homologous recombination deficiency, and knockdown of *BRCA1* or *BRCA2* markedly enhances cisplatin sensitivity in ESCC cells, suggesting the potential therapeutic relevance of *BRCA1/BRCA2* deficiency for platinum treatment in ESCC [21]. Beyond the progressive accumulation of somatic mutations, catastrophic genomic events can further promote structural genomic diversification in ESCC. For example, chromothripsis-derived double-minute chromosomes, a form of ecDNA, may drive the amplification of fibroblast growth factor receptor 1 (*FGFR1*), whereas BFB cycles can generate complex genomic rearrangements, including amplifications of *CCND1* and *MYC* [54]. Accordingly, late-stage ESCC evolution involves not only an increase in subclone number but also the acquisition of new functional programs and accelerated evolution within an established trunk architecture. This interpretation is consistent with broader analyses of late branching evolution, in which ongoing CIN generates pervasive SCNA heterogeneity and enables continuous selection and karyotype remodeling [124].

In HNSCC, analogous late evolution is reflected in the further temporal separation of point mutations and structural abnormalities [117]. *TP53* alterations are frequent in HNSCC, while activating *PIK3CA* mutations and other PI3K pathway alterations are also recurrently observed [61,118–121]. In LUSC, later events typically arise after genome doubling and include *PIK3CA* mutation, alterations in mutL homolog 1 (*MLH1*)*,* and copy number gains on 7p, 8q, and 20q; more broadly, late or subclonal drivers may affect DNA damage repair, chromatin remodeling, and other genome maintenance pathways, thereby potentially conferring greater adaptability and evolutionary potential [19]. Mutational status of *TP53* and LDL receptor-related protein 1B (*LRP1B*) has been associated with higher tumor mutational burden and greater CIN in LUSC, suggesting that late genomic states may influence therapeutic vulnerability as well as evolutionary fitness [125]. On this background, early lung squamous carcinogenesis is accompanied by aberrant basal cell clonal dynamics and progressive epithelial-state remodeling, suggesting the potential utility of lineage- and cell-state-associated features for malignant progression risk stratification [89]. In cSCC, early mutational evolution is marked by UV-associated SBS7 [47,123]. More broadly, the late temporal dynamics of SCCs can be understood as the stage at which branches acquire additional adaptive programs on top of an established trunk scaffold and, in specific contexts, undergo punctuated acceleration through ecDNA, BFB, or WGD.

### Contextual determinants that bias temporal trajectories

Importantly, the temporal trajectories described above do not unfold in isolation, although they primarily reflect tumor-intrinsic genetic and cell-state evolution. UV radiation, tobacco and alcohol exposure, viral infection, hypoxia, and oxidative stress do not replace the intrinsic temporal program, but can profoundly alter the dominant mutational processes during cancer development. In this sense, contextual factors do not merely increase mutation burden; rather, they can shape evolutionary trajectories by modifying the spectrum and activity of mutational processes [45,47].

In the esophageal epithelium, tobacco smoking and alcohol consumption increase mutation frequency and accelerate clonal expansion, particularly for mutations involving *NOTCH1* and *TP53* [12,14]. These lifestyle factors also leave characteristic genomic imprints, such as SBS16, thereby shaping the early evolutionary trajectory of ESCC [126,127]. Frequent loss of heterozygosity involving *NOTCH1* and *TP53* further suggests that biallelic inactivation of these genes contributes to clonal fitness during early ESCC evolution [101]. In addition, during progression from LGIN to HGIN, hypoxia- and reactive oxygen species (ROS)-related stress programs appear to increase [128]. More specifically, early *TP53* inactivation may create a permissive state for the subsequent accumulation of APOBEC-mediated mutations [126], whereas HGIN is characterized by the emergence of invasive and dedifferentiated epithelial cells that mark progression toward ESCC [129].

In HNSCC, viral and lifestyle-associated factors define 2 distinct temporal routes. HPV-positive tumors functionally inactivate the canonical p53 and RB tumor-suppressor pathways through the viral oncoproteins E6 and E7, whereas HPV-negative tumors more clearly retain the evolutionary imprint of tobacco- and alcohol-associated damage [52,61,114]. In cSCC, UV radiation is the prototypic temporal-shaping force: UV-associated SBS7a is detectable in keratinocytes, and early *TP53* alterations may prime premalignant progression [123], whereas later ecological remodeling of the tumor enables APOBEC-associated mutagenesis to assume a dominant role and promote invasiveness [47]. More broadly, mutational signatures can be understood as reproducible patterns of base substitutions that function as molecular footprints of specific DNA-damaging and DNA repair processes. In this framework, SBS7a/b/c/d are classically linked to UV radiation, SBS4 to tobacco exposure, and SBS2/SBS13 to dysregulated activity of endogenous APOBEC cytidine deaminases. Contextual influences thus do not simply increase the number of mutations; rather, they reshape carcinogenic trajectories by altering which events occur first, which processes dominate at particular stages, and which branches are preferentially retained.

Taken together, temporal evolution in SCCs can be conceptualized as a hierarchical and continuous process. First, early mutant clones accumulate in histologically normal epithelia and establish an abnormal competitive substrate; second, trunk drivers become progressively fixed during the premalignant stage and couple with SCNA/CIN-driven genome instability to push lesions across the threshold of malignant transformation; third, during invasive disease, subclonal branches acquire additional adaptive advantages and may undergo punctuated acceleration through ecDNA, BFB, or WGD. Meanwhile, external factors such as tobacco, alcohol, UV radiation, viral infection, hypoxia, and oxidative stress do not fundamentally alter this overall hierarchy, but they profoundly shape the specific temporal trajectories followed by different SCC types and at different stages. SCC temporal heterogeneity, therefore, should not be reduced to a simple list of repeatedly mutated genes, but instead understood as a dynamic evolutionary sequence linking competition in normal epithelium, trunk establishment, genome-scale acceleration, and stage-specific selection. A comparative overview of the early and late genomic events, spatial heterogeneity, and therapeutic implications across major SCC subtypes is provided in Table 1.

**Table 1.** Comparative spatiotemporal genomic evolution among SCC subtypes

Subtype	Early event	Late event	Spatial heterogeneity	Therapeutic implication	Reference
ESCC	Truncal mutations in *TP53* and *NOTCH1*; 11q13 amplification; *CDKN2A* deletion	Subclonal mutations in *PIK3CA*, *KIT*, *NFE2L2*, and *MTOR*; SCNA escalation	Primary-metastatic divergence; upper-biased spread; immune-linked regional selection	PI3K–mTOR or HRD-guided strategies	[20,21,83,84,88,111]
HNSCC	HPV^−^: *TP53* mutation and *CDKN2A* inactivation*;* HPV^+^: *PIK3CA* mutation and *TRAF3* loss	*PIK3CA* activation; broad SCNAs after immortalization	Node-specific branching under microenvironmental pressure	PD-1-based, subtype-guided therapy	[61,86,117–121,210]
LUSC	*NOTCH1* mutations; 3q amplification; deletions in 3p/4p/5q/17p	*PIK3CA* activation and *MLH1* inactivation; *HLA* LOH; epigenetic silencing of neoantigen genes	Mirrored subclones; immune-linked clonal heterogeneity	NGS-guided targeted/ICI combinations	[19,89,97,98,125,211]
cSCC	UV-driven *TP53/NOTCH1/FAT1*; early *TERT*p	*ARID2/MAPK* change; *APOBEC* shift	TSK invasive fronts; UV-shaped fields; fibrovascular niches	Immunotherapy; state/niche-targeted strategies	[60,82,92,93,99,107,123,228]

SCC, squamous cell carcinoma; ESCC, esophageal squamous cell carcinoma; HNSCC, head and neck squamous cell carcinoma; LUSC, lung squamous cell carcinoma; cSCC, cutaneous squamous cell carcinoma; SCNA, somatic copy number alteration; HRD, homologous recombination deficiency; PI3K, phosphoinositide 3-kinase; mTOR, mechanistic target of rapamycin; HLA LOH, human leukocyte antigen loss of heterozygosity; NGS, next-generation sequencing; TSKs, tumor-specific keratinocytes; APOBEC, apolipoprotein B mRNA editing enzyme catalytic polypeptide-like; UV, ultraviolet; *TERT*p, *TERT* promoter; HPV, human papillomavirus; PD-1, programmed cell death protein 1; ICI, immune checkpoint inhibitor; MAPK, mitogen-activated protein kinase.

## Dynamics of Epigenetic Remodeling During SCC Progression

Epigenetic modifications provide fundamental regulatory mechanisms that enable cells to respond to environmental stimuli and play pivotal roles in the precancerous evolution of SCCs. Under sustained carcinogenic pressure, epithelial cells undergo progressive epigenetic remodeling. In pre-invasive lung squamous lesions, progression-associated DNA methylation changes coincide with signatures of CIN and the transition to invasive disease [130,131]. During the transition from normal tissue to malignancy, the balance between DNA methylation and demethylation contributes to epigenetic homeostasis. Disruption of this balance generates epigenetic alterations that may be neutral, deleterious, or pro-tumorigenic. In the following sections, we examine the dynamic evolution of epigenetic mechanisms across SCC subtypes, focusing on DNA methylation, chromatin remodeling, and histone modifications [2]. These stage-associated epigenetic alterations are summarized in Fig. 2.

**Fig. 2.**
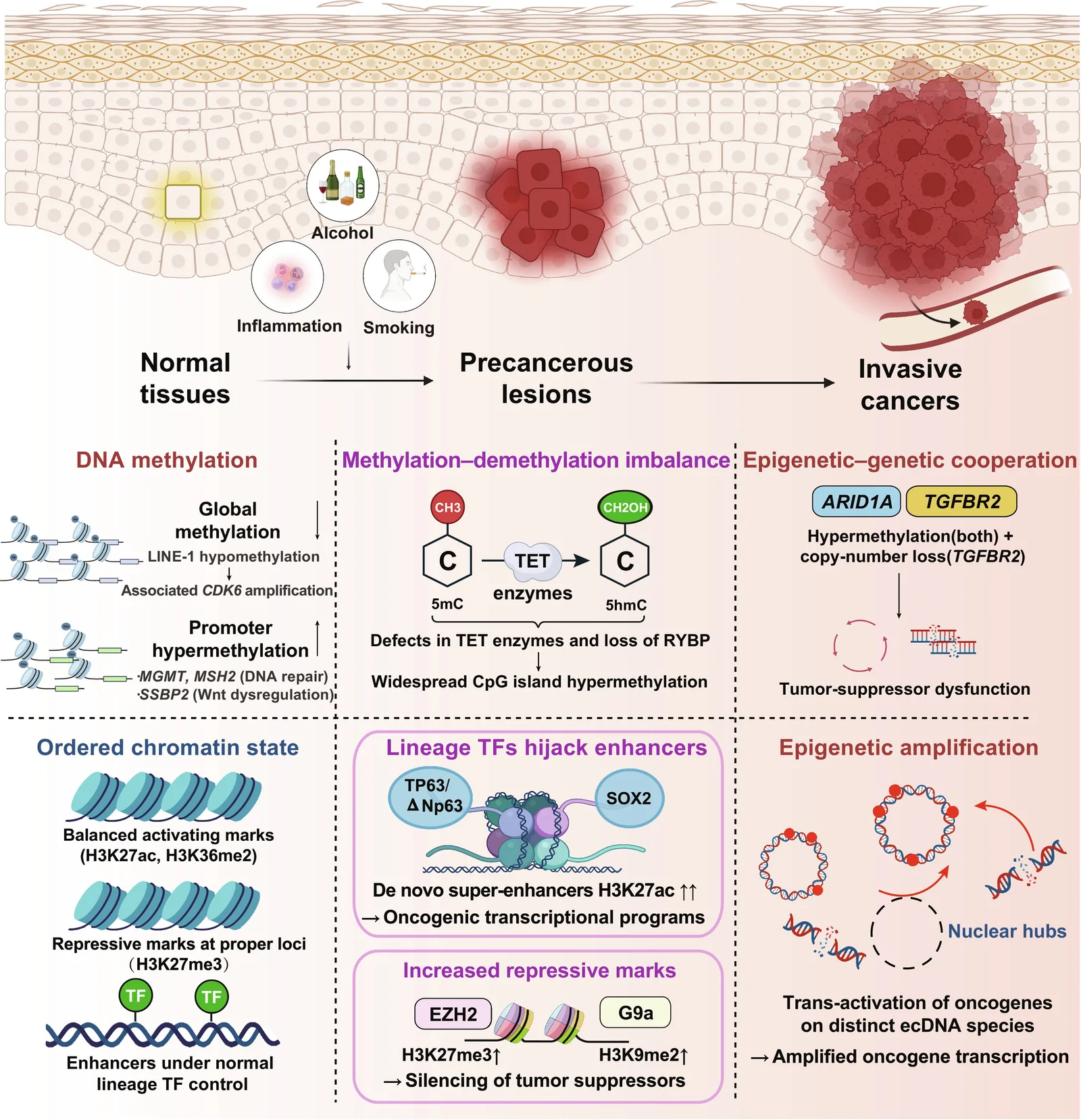
Epigenetic remodeling during squamous cell carcinoma progression. Schematic overview of the transition from normal squamous epithelium through precancerous lesions to invasive cancers. In normal tissues, DNA methylation homeostasis and an ordered chromatin state maintain lineage-specific transcription, with H3K27ac and H3K36me2 at active loci and H3K27me3 at repressed loci. During early precancerous evolution, carcinogenic exposures and inflammatory conditions, including smoking, alcohol consumption, and chronic inflammation, are associated with distinct epigenetic alterations. These include global DNA hypomethylation; LINE-1 hypomethylation associated with *CDK6* amplification; and focal promoter hypermethylation of *MGMT*, *MSH2*, and *SSBP2*, which participate in DNA repair or Wnt regulation. Defects in TET enzymes or loss of RYBP disrupt methylation–demethylation homeostasis and promote widespread CpG island hypermethylation. In parallel, TP63/ΔNp63 and SOX2 hijack lineage enhancer networks, generating H3K27ac-enriched super-enhancers that reinforce oncogenic transcriptional programs. Increased EZH2-associated H3K27me3 and G9a/EHMT2-mediated H3K9me2 further contribute to the repression of tumor-suppressive and differentiation programs. In invasive cancers, *ARID1A* promoter hypermethylation and *TGFBR2* promoter hypermethylation combined with copy number loss illustrate epigenetic and genetic–epigenetic modes of tumor-suppressor inactivation, respectively. ecDNA molecules can also assemble into nuclear hubs, enabling trans-activation of oncogenes across distinct ecDNA species and amplifying oncogenic transcription. 5mC, 5-methylcytosine; 5hmC, 5-hydroxymethylcytosine; ecDNA, extrachromosomal DNA; SCC, squamous cell carcinoma; LINE-1, long interspersed nuclear element-1; TET, ten-eleven translocation; TF, transcription factor.

### Early epigenetic remodeling in precancerous SCCs

DNA methylation is a major epigenetic mechanism that modulates chromatin accessibility and gene expression in a context-dependent manner. Methylation at promoters, enhancers, and gene bodies can exert distinct or even opposing effects on transcription. A common feature of many cancers is the coexistence of widespread genomic hypomethylation and focal hypermethylation at regulatory regions of tumor-suppressor genes. Importantly, these changes are already detectable in morphologically normal tissues and in histologically noninvasive tissue exposed to carcinogenic risk, indicating that epigenetic alterations accumulate early during carcinogenesis [132,133]. Genome-wide hypomethylation can promote CIN in experimental models [134], disrupt DNA replication timing and 3D genome organization [135], and contribute to broad transcriptional dysregulation in ESCC [136]. Hypomethylation of long interspersed nuclear element-1 (*LINE-1*) enhances cell cycle activity via up-regulation of cyclin-dependent kinase 6 (*CDK6*), thereby driving progression and invasion in ESCC and correlating with poor prognosis [137]. Promoter hypermethylation of DNA repair genes such as O^6^-methylguanine-DNA methyltransferase (*MGMT*) and mutS homolog 2 (*MSH2*) impairs repair capacity, fostering *TP53* mutations and driving ESCC initiation [138]. Similarly, hypermethylation-induced silencing of cell cycle regulators such as *CDKN2A* is frequently observed in ESCC and promotes malignant conversion [139].

In the skin, chronic UV exposure induces widespread hypomethylation in normal epidermis that persists into cSCC [140], while AK, a common precursor to cSCC, already displays cytosine–phosphate–guanine (CpG) island hypermethylation, closely resembling that of invasive lesions [141]. More broadly, active DNA demethylation contributes to epigenetic plasticity through ten-eleven translocation (TET)-dependent oxidation of 5-methylcytosine (5mC) into oxidized derivatives such as 5-hydroxymethylcytosine (5hmC) [142,143]. In SCC-related contexts, disruption of this methylation–demethylation balance may accelerate squamous transformation. In LUSC models, defects in TET methylcytosine oxidases—the enzymes responsible for 5mC oxidation—or in the Polycomb-associated protein RYBP disrupt this balance, driving widespread CpG island hypermethylation, transforming nontumorigenic bronchial epithelial cells, and enabling tumor formation in immunodeficient mice [143]. Taken together, these observations indicate that early methylation remodeling is not a secondary consequence of invasion but an early epigenetic shift that can precede malignant progression.

### Chromatin remodeling and enhancer lineage circuitry

Beyond DNA methylation, the dynamic reprogramming of histone modifications contributes to lineage plasticity and malignant state transitions across SCC subtypes. Rather than functioning as isolated covalent events, specific histone marks operate within a broader cis-regulatory circuitry, driving concerted alterations in chromatin accessibility and enhancer state remodeling [144]. A defining feature of this epigenetic rewiring is the malignant hijacking of enhancer lineage networks by master transcription factors (TFs). Master regulators such as TP63/ΔNp63 and SOX2 cooperate to activate oncogenic super-enhancers in SCC [145], while TP63 also establishes tumor-specific accessible sites and chromatin loops that reinforce squamous oncogenic programs [146]. Recent ESCC work further reinforces this coupling, demonstrating that SOX2 directly reprograms histone acetylation to rewire super-enhancer functions [147]. These lineage-specific super-enhancers are marked by high H3K27ac levels and are associated with cancer-driving transcriptional networks [148,149]. A recent pan-cancer transition atlas has reinforced this paradigm, linking changes in chromatin and enhancer accessibility to malignant state transitions and placing enhancer accessibility at the center of squamous lineage plasticity [150].

Concurrently, SCCs exploit repressive histone marks to attenuate differentiation and tumor-suppressive pathways. In ESCC, overexpression of enhancer of zeste 2 polycomb repressive complex 2 subunit (*EZH2*) is associated with elevated histone H3 lysine 27 trimethylation (H3K27me3) and poor clinical outcome, whereas overexpression of euchromatic histone lysine methyltransferase 2 (*EHMT2*) further contributes to broader dysregulation of repressive histone methyltransferase programs. Together, these alterations support a model in which repressive chromatin remodeling contributes to the attenuation of tumor-suppressive and differentiation-related programs [151–153]. In parallel, reduced histone H3 lysine 36 dimethylation (H3K36me2) represents a key chromatin state through which oncogenic histone H3 lysine 36-to-methionine substitution (H3K36M)-related signaling exerts its effects [154]. Notably, loss of nuclear receptor binding SET domain protein 1 (*NSD1*) decreases H3K36me2, induces compensatory H3K27me3, and reduces H3K27ac at cis-regulatory elements, thereby reshaping enhancer competence and immune-related transcriptional programs in SCC [155]. Similarly, nuclear receptor binding SET domain protein 3 (*NSD3*) sustains oncogenic activation in LUSC by modulating H3K36me2 [156]. A related model of squamous trans-differentiation further suggests that repression of ductal lineage programs by H3K27me3, together with TP63-driven hijacking of squamous cell fate, can enhance tumor adaptability under Wnt-deficient and hypoxic conditions [157].

Furthermore, this enhancer rewiring extends beyond the linear genome, heavily exploiting the 3D spatial topology of ecDNA [158]. Unlike chromosomal DNA, ecDNA forms hyper-accessible circular structures devoid of higher-order compaction, resulting in extensive H3K27ac deposition and a scarcity of repressive marks [159]. Critically, these ecDNAs spatially cluster into dynamic nuclear “hubs”, allowing super-enhancers on one ecDNA molecule to function in trans to activate oncogenes on distinct ecDNA species [160]. This intermolecular cooperation amplifies transcriptional output far beyond what focal amplification alone can achieve, propelling extreme tumor aggressiveness.

Beyond acetylation, the dysregulation of histone deacetylases (HDACs) further skews this transcriptional equilibrium. Loss of sirtuin 6 (*SIRT6*) enriches glycolytic cancer stem cells, enhances tumorigenic capacity, and drives aggressive SCC phenotypes [161]. In OSCC, overexpression of histone deacetylase 9 (*HDAC9*) accelerates OSCC growth by modulating cell cycle progression and inhibiting apoptosis [162]. Taken together, histone acetylation and deacetylation dynamically reshape transcriptional programs in precancerous and malignant squamous tissues by integrating histone marks, chromatin accessibility, enhancer rewiring, and lineage TF occupancy into a single regulatory program.

### Epigenetic memory and persistent state reprogramming in SCCs

Epigenetic memory refers to the stable mitotic inheritance of DNA methylation patterns and chromatin states through cell division, independent of changes in DNA sequence [163,164]. In SCC-related epithelial contexts, the evidence discussed above supports the concept that exposure- and inflammation-associated epigenetic alterations can persist in epithelial progenitor compartments and contribute to durable changes in tissue states. Such modifications can retain long-lasting traces of environmental exposure in normal epithelial tissues [165]. Crucially, these acquired marks persist through precancerous states and continue to shape tumor evolution. Epigenetic memory may promote malignant transformation by stably silencing or activating key regulatory genes in SCCs.

Precancerous lesions represent a critical transitional stage in the tumorigenesis of SCCs, typically characterized by the progressive accumulation of epigenetic alterations. In normal tissues and early lesions, such changes are often limited and focal, affecting only selected genes through DNA methylation or histone modifications. With disease progression, however, the scope and complexity of these alterations expand, ultimately driving cellular reprogramming and malignant transformation. Environmental and intrinsic stimuli act as triggers for this epigenetic remodeling. Exposures such as tobacco smoking [166], alcohol consumption [167], and chronic inflammation [168] can all induce methylation and histone modifications that progressively accumulate in normal epithelial cells. For instance, tobacco smoking can promote the hypermethylation of the single-stranded DNA-binding protein 2 (*SSBP2*) promoter, potentially contributing to Wnt pathway dysregulation and early esophageal carcinogenesis [169]. Importantly, it has been shown that epigenetic regulation alone may be sufficient to establish tumor-permissive transcriptional programs [144]. In normal esophageal tissues, ESCC risk correlates with cumulative DNA methylation alterations [170]. Similarly, alcohol consumption is associated with genome-wide DNA methylation changes in humans [167] and with distinct tumor methylation patterns in HNSCC and an independent OSCC cohort [171], while chronic inflammation modifies the DNA methylome through cytokine signaling [168].

Recent studies have clarified the molecular basis, spatial organization, and longevity of epigenetic memory in epithelial tissues. For instance, in skin epidermis, Naik et al. [132] demonstrated that transient inflammation induces persistent changes in chromatin accessibility within epithelial stem cells, establishing a long-lasting memory that primes the tissue for subsequent challenges. Extending this concept beyond the initial injury site, persistent transcriptional and chromatin priming has been identified in epidermal progenitors within distal, undamaged regions after wound resolution [172]. This memory state is characterized by sustained chromatin accessibility and reduced monoubiquitination of histone H2A at lysine 119 (H2AK119ub), which together establish an epigenetic field of cancerization that predisposes tissue to SCC onset. Importantly, the persistence of inflammatory memory in epidermal stem cells has also been linked to intrinsic DNA sequence features. In epidermal stem cells, CpG-rich memory elements coordinate DNA demethylation, H2A.Z incorporation, and methylation-sensitive TF binding, thereby helping maintain long-lived chromatin accessibility after inflammatory stress [173].

These findings suggest that repair-associated plasticity can acquire tumor-promoting properties when transient epithelial states are epigenetically stabilized. In skin and oral SCC, promoter-associated histone and DNA methylation suppress the expression of secreted frizzled-related protein 1 (*SFRP1*), thereby enhancing WNT7B-driven noncanonical Wnt signaling and c-JUN activation [174]. The resulting c-JUN–SOX2–HIF-1α axis couples cancer stemness to glycolytic reprogramming, promoting tumorigenic and metastatic potential. Together, these studies suggest that persistent epigenetic reprogramming may contribute at multiple stages of SCC pathogenesis, from establishing tumor-permissive fields to maintaining malignant stem-like states.

### Genome–epigenome reciprocity and evolutionary selection

The pathogenesis and progression of SCCs are fundamentally driven by a reciprocal feed-forward loop between genetic lesions and epigenetic remodeling. Rather than functioning in isolation, driver mutations and epigenetic alterations synergistically reinforce malignant trajectories, creating a dynamic engine for clonal evolution under selective pressure [175].

On the one hand, genetic lesions can reshape the epigenetic landscape in SCC. Specific driver mutations are often accompanied by characteristic epigenetic abnormalities. In HNSCC, *NSD1* mutations cause loss of intergenic H3K36me2, followed by DNA hypomethylation and gain of H3K27me3 [176]. In LUSC, *KMT2D* loss reprograms H3K27ac and chromatin accessibility, repressing protein tyrosine phosphatases and activating oncogenic RTK–RAS signaling [177]. In engineered murine ESCC organoids, *Sox2* overexpression together with *Trp53* and *Cdkn2a* loss promotes chromatin remodeling and expansion of the *Sox2* cistrome, reinforcing oncogenic transcriptional programs [178]. Conversely, epigenetic silencing can provide an alternative route to the inactivation of tumor suppressors and chromatin regulators. In SCC, promoter hypermethylation of AT-rich interaction domain 1A (*ARID1A*) reduces *ARID1A* expression, disrupts transcriptional homeostasis, and promotes tumor progression [179]. Epigenetic silencing can also cooperate with genetic loss, consistent with Knudson’s 2-hit model. In ESCC, promoter hypermethylation of transforming growth factor β receptor 2 (*TGFBR2*) is associated with reduced expression, whereas tumors with *TGFBR2* copy number loss show higher promoter methylation, suggesting combined genetic and epigenetic inactivation [180].

Crucially, this bidirectional reciprocity provides the extreme adaptability required for tumor cells to survive external evolutionary bottlenecks, such as systemic therapy and metastasis. Under therapeutic pressure, tumor cells may acquire resistance through genetic mutations, copy number variations, or epigenetic reprogramming. For instance, targeted therapies can trigger the rapid transcriptional derepression of the cytidine deaminase *APOBEC3A*, fueling ongoing mutagenesis and driving the expansion of resistant subclones via DNA double-strand breaks in lung cancer [181].

In metastatic tumors, heterogeneity in actionable genomic alterations is often limited across serial biopsies. Longitudinal whole-genome sequencing of 231 patients with metastatic solid tumors revealed that genomic evolution under therapeutic pressure was limited, particularly for actionable biomarkers. This suggests that a single sequencing analysis may be sufficient to guide therapeutic decision-making in many patients with metastatic disease [182]. In summary, the seamless integration of epigenetic memory with genomic instability establishes a robust adaptive landscape. This genome–epigenome reciprocity empowers SCC clones to circumvent intrinsic barriers such as senescence, resist therapeutic eradication, and relentlessly propel metastatic relapses.

## Clinical Implications of Genetic and Epigenetic Alterations in SCC Evolution

Genetic and epigenetic alterations are central features of SCC evolution and have important translational implications for early interception, progression control, and therapeutic optimization. Across SCC subtypes, including esophageal, cutaneous, lung, and oral SCCs, the transition from normal epithelium to precancerous lesions and ultimately to invasive cancer generally follows a stepwise but molecularly heterogeneous trajectory [183–185]. This evolutionary continuum provides a framework for identifying when lesions acquire molecular features associated with malignant commitment, when diversification accelerates, when immune escape becomes clinically relevant, and when intervention may be most effective. Because the strength of mechanistic and clinical evidence remains uneven across SCC subtypes, the discussion below combines shared evolutionary principles with selective site-specific examples and potential clinical applications. In this context, the translational implications of SCC evolution can be organized into 4 interrelated dimensions: tumor initiation and interception, progression and malignant diversification, immune surveillance and immune evasion, and therapeutic response and resistance.

### Implications for tumor initiation and interception

The precancerous phase of SCCs may represent the most actionable stage of disease evolution, because many driver-associated and epigenetically primed alterations are already established before overt invasion occurs. In ESCC, genomic comparisons between precursor lesions and matched carcinomas have shown that intraepithelial neoplasia can already harbor mutational spectra and CNA profiles resembling those of invasive tumors rather than simple hyperplasia [186]. These observations suggest that, in at least a subset of lesions, molecular commitment may precede conventional histopathologic transition and may therefore provide an earlier basis for risk stratification. This interpretation is further supported by a population-based study integrating endoscopic iodine staining, genomic profiling, and epidemiology, which showed that Lugol iodine nonstaining mucosa without histological dysplasia already accumulated substantial clonal expansions, mutations, and CNAs, together with an increased long-term risk of malignant progression [187]. Collectively, these findings suggest that surveillance and interception strategies may need to extend upstream, from overt cancer and histologically defined dysplasia to molecularly advanced precursor fields.

The translational relevance of this early window is further supported by evidence at the cellular and ecosystem levels. Recent studies suggest that emerging lesions can remodel the surrounding precancerous niche at early stages, and that this niche remodeling may contribute to nascent tumor persistence [76,188]. Accordingly, early interception may need to target not only high-risk epithelial clones but also the stromal and immune context that permits their persistence before overt invasion [76]. In early lung squamous carcinogenesis, a transitional basal cell state has been identified before overt malignancy, supporting the use of epithelial cell-state changes as potential indicators of premalignant progression [89]. At the tissue scale, single-cell and spatial transcriptomic studies indicate that epithelial reprogramming and microenvironmental remodeling are detectable in both low- and high-grade precursor lesions, providing potential cellular and spatial markers for early risk assessment [189–191]. In ESCC, epithelial communication emerges in early precursor lesions and intensifies with progression, further supporting the potential value of molecular monitoring before invasion [192]. Similarly, in OSCC, precancerous epithelial cells already communicate with vascular, fibroblast, and myeloid cells, indicating that stromal support may become established before invasion [191]. Taken together, these observations suggest that SCC interception should not be understood simply as earlier treatment of established cancer, but as the risk-guided identification and interruption of biologically advanced precursor lesions before invasive diversification becomes established.

### Implications for progression and malignant diversification

Progression from precancerous lesions to invasive SCC is driven not only by the accumulation of additional genetic lesions but also by increasing CIN, cell-state transitions, and stromal cooperation, all of which collectively enable malignant diversification. Integrative analyses of multistage squamous carcinogenesis have shown that the transition from benign precursors to malignant lesions is accompanied by a shift from diploid to aneuploid epithelial populations, particularly at the high-grade intraepithelial stage, suggesting that aneuploidy may help identify lesions approaching malignant transition [193]. Copy number instability is not merely a by-product of progression, but may mark a key transition in malignant evolution. More broadly, CIN may help distinguish indolent precursor lesions from those acquiring the plasticity required for invasive growth. WGD may further identify lesions with an increased capacity to tolerate genomic disruption and undergo subsequent diversification [122]. In this sense, progression in SCCs may be better understood as a transition in evolutionary capacity rather than as a purely linear increase in mutational burden, with implications for molecular risk stratification and longitudinal monitoring.

This malignant transition is further stabilized by nongenetic reprogramming and cell-state plasticity. Although the mechanistic basis of these epigenetic changes is discussed above, their key implication here is that epigenetic remodeling can lock evolving squamous cells into states that are more permissive for invasion and diversification [150,175]. Accordingly, clinically relevant progression is likely to reflect the coupling of genomic instability with persistent aggressive cell states and support, rather than genetic change alone. Single-cell studies of ESCC illustrate that genomic diversification can coexist with immunosuppressive microenvironment states, although the causal relationships between these features remain to be established [194].

As lesions progress, increasing stromal engagement may provide an additional indicator that a precursor ecosystem is becoming permissive for invasion. Multistage single-cell and spatial studies across SCCs indicate that fibroblast, endothelial, and myeloid cells increasingly participate as lesions advance, forming permissive niches that support growth and invasion [77,190,195–198]. In ESCC, tumor-driven fibroblast reprogramming provides a representative example of how stromal changes can stabilize a permissive and immunosuppressive niche during progression [128]. In cSCC, spatially organized interactions between TSKs and stromal cells similarly identify invasive-front niches associated with progression [82]. Altogether, these observations suggest that progression blockade will require strategies targeting both cell-intrinsic instability and cell-extrinsic reinforcement. The progression phase of SCC evolution therefore represents a critical translational interval for risk-adapted intervention, particularly before highly heterogeneous invasive ecosystems become entrenched.

### Immune surveillance and immune evasion in SCC evolution

Immune pressure functions as a dynamic evolutionary filter throughout SCC development, shaping which premalignant clones are eliminated, which persist, and which ultimately escape and expand. Evidence from early squamous carcinogenesis indicates that immune remodeling can occur before invasion, challenging the traditional view that immune escape is purely a late-stage event [185,190,197,198]. For example, in lung squamous carcinogenesis, immune reprogramming emerges at the high-grade dysplasia stage, with activated T cell signatures coexisting with expanded neutrophil and macrophage populations [185]. These findings indicate that immune selection is already active during precancerous progression, consistent with the cancer immunoediting framework of elimination, equilibrium, and escape. Premalignant clones that evade elimination may persist in an equilibrium state, during which continued immune pressure can favor the emergence and expansion of immune-evasive populations [199]. This early phase of immune engagement may therefore provide a window for immune-based risk stratification and interception before escape mechanisms become stably established.

As SCC evolves, immune evasion becomes increasingly embedded within clonal evolution, with consequences for lesion persistence, progression risk, and subsequent therapeutic responsiveness. Across SCCs and related squamous malignancies, shared immune-evasive mechanisms include impaired antigen presentation, HLA loss of heterozygosity, enrichment of dysfunctional or exhausted T cells, and suppressive stromal and myeloid niches, often accompanied by regulatory T cell (Treg) accumulation [83,190,197]. Epigenetic remodeling can further stabilize these states. For example, HPV-encoded circE7 in HNSCC suppresses *H3K27* acetylation at the galectin 9 (*LGALS9*) promoter [200], whereas IFNα-induced lncMX1-215 disrupts GCN5-mediated H3K27 acetylation and reduces PD-L1 and galectin-9 expression in HNSCC [201]. In ESCC, accumulating evidence indicates that immune infiltration imposes selective pressure during tumor evolution. Spatial heterogeneity in *HLA* and β2-microglobulin (*B2M*) gene expression has been linked to regional differences in CD8^+^ T cell and natural killer (NK) cell infiltration, supporting the notion that local immune pressure promotes the selection of immune-evasive clones with impaired antigen presentation and HLA loss [83]. Complementary analyses indicate that the immunoediting score may outperform absolute CD8^+^ T cell abundance in capturing immune selection and predicting prognosis in ESCC [88]. These findings support biomarker frameworks that integrate immune-cell functional state, antigen presentation capacity, and spatial context rather than relying on immune-cell density alone. Spatial and single-cell studies further suggest that immune risk assessment should incorporate stromal and myeloid context, which can sustain immune exclusion during progression from precursor lesions to invasive tumors [95,129]. Taken together, these observations support the use of immune escape as a clinically relevant dimension of SCC evolution for assessing lesion persistence, invasive progression risk, and future therapeutic responsiveness. Intervention may be most effective while high-risk lesions remain in an immune equilibrium state, before antigen presentation defects and immune-excluded niches become fixed. The key determinants, representative mechanisms, immune consequences, and potential therapeutic implications of the SCC immune microenvironment are summarized in Table 2.

**Table 2.** Key determinants of the immune microenvironment in squamous cell carcinoma

Determinant	SCC context	Immune consequence	Mechanistic basis	Potential therapeutic implication	Reference
HLA-I/B2M axis (antigen presentation dysfunction)	Treatment-naive ESCC and early-stage NSCLC, including LUSC; multiregional or subclonal heterogeneity	Impaired CD8^+^ T cell and NK cell recognition	*HLA* LOH, *B2M* heterogeneity, and neoantigen loss or silencing	Checkpoint-based combinations and restoration of antigen presentation	[83,88,98]
*KMT2C/MLL3* loss (epigenetic immune regulation)	Upper aerodigestive SCC; early clonal event	Reduced cytotoxic T cell infiltration	Loss of MLL3–GRHL2–IRF1 signaling reduces *CXCL9/CXCL10* expression	Potential stratification for ICB response and epigenetic targeting	[205]
*NSD1* loss (histone methylation dysregulation)	*NSD1*-deficient HNSCC; immune-cold state	Reduced immune cell recruitment	Loss of H3K36me2 and gain of H3K27me3 repress innate immune-response genes	EZH2-directed restoration of immune infiltration	[155,233]
HPV16-encoded circE7 (viral-epigenetic regulation)	HPV16-positive HNSCC	Reduced CD8^+^ T cell infiltration and function	circE7-mediated ACC1 activation reduces H3K27ac at the *LGALS9* promoter and decreases galectin-9 expression	Potential combined anti-PD-1/anti-TIM-3 blockade	[200]
IFNα-lncMX1-215-GCN5 axis (epigenetic checkpoint regulation)	HNSCC	Reduced PD-L1 and galectin-9 expression and attenuation of local immunosuppression	lncMX1-215 disrupts GCN5-mediated H3K27ac at immune-checkpoint-associated loci	Potential epigenetic modulation of immune-checkpoint expression	[201]
TDO2^+^ myofibroblast (metabolic stromal regulation)	Oral leukoplakia-to-OSCC malignant transformation	Treg induction and CD8^+^ T cell dysfunction	TDO2-dependent tryptophan catabolism creates a locally immunosuppressive niche	TDO2 inhibition and stromal/metabolic immunomodulation	[190]
Fibroblast-defined immune niche (spatial stromal regulation)	Established HNSCC; HPV-positive immune-hot and HPV-negative immune-cold ecosystems	Immune-hot versus immune-cold divergence	FRC-like fibroblasts are enriched in immune-hot regions, whereas immunosuppressive CAF populations characterize immune-cold regions	Niche-stratified immunotherapy and stromal targeting	[202]

HLA-I, human leukocyte antigen class I; *B2M*, β2-microglobulin; CD8, cluster of differentiation 8; NK, natural killer; ICB, immune checkpoint blockade; TDO2, tryptophan 2,3-dioxygenase; FRC, fibroblastic reticular cell; *LGALS9*, galectin-9; SCC, squamous cell carcinoma; ESCC, esophageal squamous cell carcinoma; NSCLC, non-small cell lung cancer; LUSC, lung squamous cell carcinoma; HLA LOH, human leukocyte antigen loss of heterozygosity; HNSCC, head and neck squamous cell carcinoma; HPV, human papillomavirus; ACC1, acetyl-CoA carboxylase 1; PD-1, programmed cell death protein 1; TIM-3, T-cell immunoglobulin and mucin-domain-containing protein 3; IFNα, interferon α; PD-L1, programmed cell death ligand 1; OSCC, oral squamous cell carcinoma; Treg, regulatory T cell; CAF, cancer-associated fibroblast.

### Implications for immune response and therapeutic resistance

Building on this evolutionary framework, the dynamics of immune surveillance and immune escape have direct implications for therapeutic response and resistance in SCCs. The coupling between tumor cell plasticity and immune escape may help explain heterogeneous responses to immunotherapy. Primary resistance may, in part, reflect preexisting local immune ecosystem states rather than tumor-intrinsic properties alone. This interpretation is supported by spatial and single-cell analyses in HNSCC, showing that immune-hot and immune-cold states are associated with distinct fibroblasts, lymphoid, and myeloid ecosystems [202]. Such spatial ecosystem states may help identify tumors unlikely to respond to immune checkpoint blockade without additional stromal or myeloid modulation. In such settings, early detection alone may not translate into therapeutic responsiveness unless the underlying immune-excluded state is also identified and addressed. By contrast, acquired resistance emerges when therapeutic pressure reshapes clonal architecture and cell states over time [181,203]. Treatment-driven selection and lineage plasticity may thereby undermine response durability [204]. Accordingly, resistance can be viewed as a staged evolutionary process in which preexisting drivers, adaptive responses to therapeutic stress, and late lineage plasticity together shape distinct resistance trajectories that may require different therapeutic strategies [204]. Loss of the *KMT2C* (histone methyltransferase MLL3), a frequent early clonal event in upper aerodigestive tract SCCs, illustrates how early molecular states may preconfigure subsequent immunotherapy response. MLL3 loss disrupts T cell-recruiting chemokine signaling, impairs cytotoxic T cell infiltration, and promotes resistance to immune checkpoint blockade therapy [205]. Taken together, these observations suggest that immune responsiveness in SCCs should be interpreted as an evolving property of precursor lesions and tumors, rather than a static baseline feature.

From a therapeutic perspective, these findings provide a rationale for evolution-informed combination strategies beyond single-agent immunotherapy. Epigenetic therapies, stromal modulation, and rational combinations with immune checkpoint blockade may be especially valuable when matched to the pretreatment and evolving biological state of the lesion or tumor [155,190,206,207]. Among epigenetic approaches, DNA methyltransferase inhibitors are of particular interest because evidence from other malignancies supports their therapeutic potential [208,209], although their application in solid tumors remains at an early stage [206]. More broadly, HNSCC also exhibits subtype-specific therapeutic vulnerabilities that may inform treatment selection. HPV status may help define distinct therapeutic vulnerabilities [210]. Therapeutic goals should therefore be stage-specific: In precursor or early invasive lesions, intervention should aim to prevent immune escape from becoming fixed, whereas in advanced SCCs, combination strategies may be required to target immune suppression, state plasticity, and clonal adaptability simultaneously. Beyond informing therapeutic combination strategies, evolutionary profiling may facilitate precision treatment selection. Molecular profiling may also uncover actionable alterations that support genotype-guided treatment selection in clinically challenging SCCs [211]. Accordingly, immune-oriented intervention in SCCs should be stage-aware, state-aware, and evolution-aware, rather than being applied uniformly across all lesions or guided by a single baseline biomarker [212].

## Early Detection, Biomarkers, and Interception

### Challenges in early SCC detection

Early detection of SCCs remains challenging not only because early lesions are often clinically inconspicuous but also because biological heterogeneity, sampling limitations, and implementation barriers jointly reduce screening performance. Early-stage SCCs are frequently asymptomatic, leading to delayed diagnosis and poorer outcomes [213]. Conventional tissue biopsy remains invasive, costly, and heterogeneous in diagnostic yield, limiting its scalability for population-level screening [214]. In addition, suboptimal participation in screening programs, delays in diagnostic confirmation, and insufficient follow-up of screen-positive individuals can all weaken the effectiveness of early detection strategies [215]. These limitations are especially relevant in high-incidence settings, where attrition across the screening pathway may offset the potential benefit of technically feasible screening tools.

Beyond these practical barriers, early SCC detection is also biologically difficult. Precancerous lesions are heterogeneous, often release weak or inconsistent molecular signals, and may not be adequately represented by single-site tissue sampling. Clinical specimens from the earliest disease stages are relatively scarce, and biomarkers with sufficient sensitivity, specificity, and lesion-level risk discrimination remain limited [216]. Therefore, the challenge of early SCC detection is not merely technical; it arises from the combined effects of low-abundance signals, lesion heterogeneity, and implementation constraints. This is why biomarker development for SCCs is increasingly moving from one-dimensional assays toward biology-informed and multimodal strategies.

### Emerging biomarker classes for precancerous and early SCC detection

Recent studies have expanded the biomarker landscape for precancerous and early SCC detection from single analyses to multiple molecular classes, each capturing different dimensions of early tumor evolution. Among the most promising are circulating nucleic acid biomarkers. In ESCC, methylated cell-free DNA (cfDNA) markers such as otopetrin 2 (*OTOP2*) and potassium voltage-gated channel subfamily A member 3 (*KCNA3*) are significantly elevated in patients with HGIN and ESCC, and simplified cell-free DNA methylation assays targeting these loci have shown strong diagnostic performance, with high sensitivity and specificity in multicenter validation studies [212,217]. Similarly, cfDNA-based copy number markers, including *PIK3CA, SOX2*, epidermal growth factor receptor (*EGFR*), *MYC*, and *CCND1*, have shown potential utility for early ESCC screening [218]. These approaches are attractive because they directly capture molecular abnormalities. However, copy number patterns may be shared across SCCs from different anatomical sites, which may limit organ specificity and complicate lesion attribution in pan-SCC settings.

Additional biomarker classes may further complement circulating DNA assays. Autoantibody-based models, such as the plsRglm quadruplicate autoantibody model, have shown the capacity to identify esophageal precancerous lesions and to detect a subset of individuals who subsequently developed ESCC several months to years before clinical diagnosis [219]. Metabolite-based biomarkers are also emerging: Pipecolic acid, for example, demonstrated strong discriminatory performance between dysplastic lesions and controls in a 4-nitroquinoline 1-oxide (4-NQO)-induced ESCC model, although its predictive value in human precursor lesions remains to be further established [220]. Likewise, microbiome- and proteome-based profiling may provide ecosystem-level biomarkers, as illustrated in squamous epithelial precancerous lesions such as anal intraepithelial neoplasia [221].

Saliva-based biomarkers represent another particularly attractive class because of their noninvasive and repeatable sampling properties. In ESCC, a salivary extracellular vesicle and particle (EVP)-based 6-microRNA signature demonstrated high accuracy for early-stage detection and prognostic stratification [222]. In OSCC, saliva contains a broad spectrum of biomarker candidates spanning the genome, transcriptome, proteome, metabolome, immunome, and microbiome [223]. Taken together, these findings suggest that no single biomarker class is likely to be sufficient across all SCC contexts; rather, complementary biomarker classes will be needed to capture early clonal, epigenetic, metabolic, and microenvironmental changes (Table 3).

**Table 3.** Biomarker classes for precancerous and early SCC detection

Biomarker class	Representative example	Biological layer	Strength	Clinical implication	Reference
cfDNA methylation	*KCNA3* and *OTOP2*	Epigenetic	Sensitive for HGIN and early ESCC	Early detection	[212,217]
cfDNA copy number alteration	*PIK3CA, SOX2, EGFR, MYC,* and *CCND1*	Genomic	Reflects clonal expansion and instability	Risk stratification	[218]
Autoantibody signature	plsRglm model	Immune/systemic	May detect lesions before diagnosis	Complementary screening	[219]
Metabolite biomarker	Pipecolic acid	Metabolic	Reflects functional reprogramming	Experimental detection	[220]
Microbiome-derived biomarker	Anal lesion-associated profile	Ecosystem-level	Captures microenvironmental alterations	Complementary biomarkers	[221]
Saliva-based biomarker	EVP-derived 6-miRNA signature	Local multi-omics	Noninvasive and repeatable	Screening and monitoring	[222]
Saliva multi-omics	OSCC saliva profile	Integrative local multi-omics	Broad biological coverage	Detection and surveillance	[223]

cfDNA, cell-free DNA; *KCNA3*, potassium voltage-gated channel subfamily A member 3; *OTOP2*, otopetrin 2; HGIN, high-grade intraepithelial neoplasia; ESCC, esophageal squamous cell carcinoma; *PIK3CA*, phosphatidylinositol-4,5-bisphosphate 3-kinase catalytic subunit α; *SOX2,* SRY-box transcription factor 2; *EGFR*, epidermal growth factor receptor; *MYC*, MYC proto-oncogene; *CCND1*, cyclin D1; EVP, extracellular vesicle and particle; miRNA, microRNA; OSCC, oral squamous cell carcinoma

### Multimodal early interception strategies

Because precancerous and early SCC lesions generate weak, heterogeneous, and context-dependent signals, multimodal strategies are likely to outperform single-modality assays in both detection and risk stratification. Emerging technologies are beginning to address this challenge by amplifying low-abundance tumor-associated signals and integrating multiple layers of biological information. For example, a CRISPR/Cas12a-based detection platform enhanced by spherical gold nanoparticles and DNA linkers has achieved highly sensitive detection of low-abundance tumor proteins and substantially improved ESCC diagnosis in multicenter settings [224]. Such platforms illustrate how technical innovation can partly compensate for the inherently low signal intensity of early lesions.

Artificial intelligence (AI) is further expanding the scope of early SCC detection. Machine learning models integrating cytological and epidemiological features from high-risk populations have shown strong performance in predicting HGIN and ESCC before endoscopy [225]. Likewise, stacked ensemble models based on cfDNA fragmentomic features have achieved high sensitivity and specificity even under low-coverage sequencing conditions, supporting the feasibility of cost-efficient liquid biopsy-based screening [226]. AI-assisted endoscopy has also improved detection rates for superficial ESCC and precancerous lesions in randomized multicenter studies [227]. Comparable AI-driven approaches are being explored in cSCC and other SCCs to improve lesion classification, reduce unnecessary biopsies, and accelerate referral or treatment [228,229]. However, the greatest translational value may lie not in any single AI tool, but in their ability to serve as integrative layers across imaging, pathology, and liquid biopsy.

The most promising direction, therefore, is multimodal early interception rather than single-test early detection. A representative example is the expanded multi-modal analysis (EMMA) framework, which integrates cfDNA methylation, CNAs, and fragmentation features from whole-genome bisulfite sequencing and markedly improves ESCC detection accuracy [230]. Conceptually, such models are important because they move the field beyond asking whether a lesion is present, toward determining whether a lesion is biologically likely to progress and therefore warrants intensified surveillance or active interception. Multiregion evidence further supports a state-aware view of interception. For example, immune-infiltrated and sparsely infiltrated NSCLC regions showed distinct routes of neoantigen escape, including HLA loss, copy-number loss, and promoter hypermethylation, highlighting the value of biologically stratified intervention [98]. Future progress will depend on combining multi-omics assays, AI-assisted interpretation, and clinically deployable workflows in prospective cohorts. More broadly, recent precancer-focused frameworks have argued that effective interception will require not only earlier detection but also precision remodeling of the precancer microenvironment in parallel with biomarker-guided risk assessment [231]. Ultimately, multimodal interception strategies may help redefine SCC screening from a passive search for existing cancer to an active effort to identify and interrupt molecularly advanced precursor lesions before invasive transformation occurs (Table 4).

**Table 4.** Interception strategies mapped to tumorigenesis, progression, and immune evasion

Evolutionary stage	Key features and mechanism	Interception strategy	Clinical goal	Reference
Tumorigenesis	Mutated epithelial clones and early molecular alterations in histologically normal or minimally altered tissues, driven by clonal expansion and early molecular commitment	Identify and monitor high-risk epithelial fields through surveillance and preventive monitoring	To delay or prevent malignant commitment	[76,186,187]
Precancerous progression	Progressive molecular and ecosystem remodeling in advanced precursor lesions, characterized by increasing genomic instability, evolving cell states, and stromal engagement	Intervene in biologically advanced precursor lesions using local treatment and molecular monitoring	To reduce progression to invasive SCC	[122,193]
Malignant diversification	Branching evolution and progressively fixed aggressive cell states supported by genomic instability, epigenetic stabilization, and stromal cooperation	Intercept early diversification before ecosystem stabilization	To limit invasion and heterogeneity	[153,190,196]
Early immune engagement	Immune editing and activated T cell responses in high-risk lesions, corresponding to the elimination and equilibrium phases of immunoediting	Strengthen immune surveillance with immunomodulatory strategies	To promote immune-mediated clearance	[185,199]
Immune evasion phase	HLA loss, PD-L1 up-regulation, exhausted T cells, and suppressive myeloid/Treg niches caused by antigen presentation defects and checkpoint activation	Reverse immune suppression with checkpoint-based combination strategies	To improve immune responsiveness	[97,98,129]
Advanced/resistant SCC	Plasticity, immune exhaustion, and therapy-adapted tumor states driven by therapy-selected clonal evolution and adaptive resistance	Apply biomarker-guided or mechanism-guided combination strategies to overcome therapy-adapted resistant states	To help overcome resistance and prolong response	[203]
Detection-guided interception	Multimodal biomarker positivity and risk stratification enabled by integration of methylation, copy number, fragmentomics, imaging, and AI	Escalate surveillance or consider intervention according to biological risk using multimodal detection frameworks	To support a shift from early detection toward early interception	[226,227,230]

SCC, squamous cell carcinoma; HLA, human leukocyte antigen; PD-L1, programmed cell death ligand 1; Treg, regulatory T cell; AI, artificial intelligence

## Conclusions and Future Perspectives

SCCs of the esophagus, head and neck, lung, and skin are increasingly understood not as lesions that arise abruptly at the point of histological malignancy but as the outcome of a prolonged evolutionary process unfolding across altered epithelial fields [10–15]. Across these anatomically distinct tissues, current evidence supports a shared framework in which mutant clonal expansion, genome-scale instability, epigenetic remodeling, and local ecological selection jointly shape progression from histologically normal epithelium to precancerous lesions and invasive carcinoma [3,5,83,88,111,129,175]. In this sense, SCC pathogenesis is more appropriately viewed as a spatiotemporally organized continuum than as a sequence of isolated stage-specific events [10,11].

This perspective also suggests that the most informative biological boundary in SCCs may not always coincide with conventional histopathological categories. Rather, malignant potential may become established when altered epithelial fields acquire sufficient genomic plasticity, epigenetic reinforcement, and microenvironmental support to sustain further progression [111,144,175,186,187]. Temporal ordering and spatial organization should therefore be interpreted not as parallel descriptive dimensions but as complementary readouts of the same evolving system [9,20,88,129]. Within this system, genetic alteration, chromatin and DNA methylation change, immune pressure, and stromal remodeling appear to interact in ways that influence both the pace and direction of disease evolution [3,83,128,155,184,205].

A practical implication of this framework is that opportunities for intervention may arise earlier than is often assumed. If biologically consequential changes are already present in advanced precursor fields, then the clinical objective may need to extend beyond earlier diagnosis of invasive cancer toward earlier recognition and interruption of high-risk evolutionary trajectories [76,187,230–232]. This shift has implications for lesion classification, surveillance, biomarker development, and therapeutic prioritization [212,216,217,228–230]. At the same time, in established tumors, an evolutionary framework may help explain why therapeutic response is often regionally and temporally heterogeneous, and why durable benefit may depend on strategies that address clonal adaptability, epigenetic plasticity, and microenvironmental support together rather than separately [155,181,202,203,205–210].

Future progress will likely depend on longitudinal and spatially resolved studies that define the tipping points of malignant commitment, multimodal biomarkers that capture both molecular alteration and ecological context, and mechanistic work clarifying how genome–epigenome reciprocity is coupled to tissue damage, immune selection, and stromal cooperation [76,129,175,185,202,230,231]. More broadly, advancement in SCC research may increasingly rely on shifting from descriptive cataloguing toward evolution-informed prevention, risk stratification, and interception [216,230,231]. A spatiotemporal view of SCC pathogenesis may therefore offer a more coherent basis for understanding disease continuity and for guiding earlier and more precise clinical intervention.
